# LRRK2 Transport Is Regulated by Its Novel Interacting Partner Rab32

**DOI:** 10.1371/journal.pone.0111632

**Published:** 2014-10-31

**Authors:** Dieter Waschbüsch, Helen Michels, Swantje Strassheim, Edith Ossendorf, Daniel Kessler, Christian Johannes Gloeckner, Angelika Barnekow

**Affiliations:** 1 Department of Experimental Tumorbiology, Westfälische Wilhelms University Muenster, Muenster, Germany; 2 Research Unit Protein Science, Helmholtz Zentrum München, Neuherberg, Germany; 3 Medical Proteome Center, Institute for Ophthalmic Research, Eberhard Karls University Tübingen, Tübingen, Germany; University of Pittsburgh, United States of America

## Abstract

Leucine-rich repeat kinase 2 (LRRK2) is a multi-domain 280 kDa protein that is linked to Parkinson's disease (PD). Mutations especially in the GTPase and kinase domains of LRRK2 are the most common causes of heritable PD and are also found in sporadic forms of PD. Although the cellular function of LRRK2 is largely unknown there is increasing evidence that these mutations cause cell death due to autophagic dysfunction and mitochondrial damage. Here, we demonstrate a novel mechanism of LRRK2 binding and transport, which involves the small GTPases Rab32 and Rab38. Rab32 and its closest homologue Rab38 are known to organize the trans-Golgi network and transport of key enzymes in melanogenesis, whereas their function in non-melanogenic cells is still not well understood. Cellular processes such as autophagy, mitochondrial dynamics, phagocytosis or inflammatory processes in the brain have previously been linked to Rab32. Here, we demonstrate that Rab32 and Rab38, but no other GTPase tested, directly interact with LRRK2. GFP-Trap analyses confirmed the interaction of Rab32 with the endogenous LRRK2. In yeast two-hybrid experiments we identified a predicted coiled-coil motif containing region within the aminoterminus of LRRK2 as the possible interacting domain. Fluorescence microscopy demonstrated a co-localization of Rab32 and LRRK2 at recycling endosomes and transport vesicles, while overexpression of a constitutively active mutant of Rab32 led to an increased co-localization with Rab7/9 positive perinuclear late endosomes/MVBs. Subcellular fractionation experiments supported the novel role of Rab32 in LRRK2 late endosomal transport and sorting in the cell. Thus, Rab32 may regulate the physiological functions of LRRK2.

## Introduction

LRRK2 is a multi-domain protein of 280 kDa that contains both, a GTPase domain (Ras of Complex = ROC) and a kinase domain, regulating its cellular function [1], [2], . Mutations especially in these GTPase and kinase domains of LRRK2 are the most common causes of heritable Parkinson's disease (PD) and also found in sporadic forms of PD [4]. The cellular function of LRRK2 remains unknown, although there is evidence that these mutations lead to cell death due to autophagic dysfunction and mitochondrial damage [5], [6], [7].

LRRK2 has several known direct interacting partners, including a couple of small GTPases from the Ras superfamily. The first GTPase shown to directly interact with LRRK2 was Rab5B, linked to the regulation of synaptic vesicle endocytosis [8]. Rac1 and CDC42 were also identified as LRRK2 interacting partners [9], [10]. In contrast to Rab5B, Rac1 is able to bind LRRK2 in a nucleotide independent manner, but the activity status strongly influences LRRK2 localization within the cell. Furthermore, the constitutively active Rac1 mutant is able to rescue the neurite retraction effect of the LRRK2 mutation G2019S in SH-SY5Y cells [9]. In *Drosophila* it has been demonstrated that the homologue to LRRK2 directly binds to Rab7 thus suggesting a role in late endosomal/multivesicular body (MVB) or lysosomal pathways [11]. The authors showed that the *Drosophila* homologue of the most common PD mutant G2019S displays increased co-localization with Rab7. In addition to *Drosophila*, human brain and cultured human cells reveal LRRK2 localization to late endosomal or lysosomal compartments [12], [13], [14]. Recent studies show that Rab7L1 (or Rab29), the Rab protein most closely related to Rab32 and Rab38, is a LRRK2 interacting protein as well [15], [16].

In this study, we demonstrate a novel mechanism of LRRK2 binding and transport, which involves the small GTPase Rab32. Rab32 and its closest homologue Rab38 are known to organize the trans-Golgi network (TGN) and thereby the transport of key enzymes in melanogenesis like tyrosinase and Tyrp1 [17]. This transport is mediated by the first known Rab32 and Rab38 effector Varp (also known as ANKRD27) [18]. This protein was known as a guanine nucleotide exchange factor (GEF) for Rab21 and involves the SNARE protein Vamp7 in Tyrp1 trafficking [19], [20].

Recently, novel Rab32 interacting partners were identified and their role in melanogenesis and the generation of other types of lysosome related organelles (LRO) was investigated [21]. LROs are organelles sharing common features of lysosomes but with a different specialized function. The group of LRO includes melanosomes, lytic granules in T-cells, lamellar bodies in aveolar endothelial cells or the dense granule in platelets [22], [23]. It could be demonstrated that Rab32 influences endosomal trafficking by direct interaction with adapter protein complexes AP-1, AP-3 and BLOC-2 (biogenesis of lysosome related organelles complex) [24]. The AP-3 complex was also shown to play a role in dense granule biogenesis [25]. The protein complex BLOC-3, which is a GEF for Rab32 and Rab38, also plays a role in LRO biogenesis. Furthermore, one of the proteins in this complex is mutated in the Hermansky-Pudlak syndrome [26].

However, the role of Rab32 in non-melanogenic cells is poorly understood yet. It was shown, that Rab32 is a PKA anchoring protein (AKAP) in melanogenic and non-melanogenic cells [27], [28]. In contrast to the finding that Rab32 is located to mitochondria in WI-38 fibroblasts, HeLa and COS7 cells, no co-localization of Rab32 with mitochondria could be detected in melanocytes and some other cell lines by other groups [17], [29]. Furthermore, there is evidence for Rab32 being involved in autophagy and phagocytotic digestion of bacteria [29], [30], [31]. A recent study implicates Rab32 in brain inflammation processes - Rab32 and Rab20 mRNA levels were increased upon lipopolysaccharide (LPS) injection in mouse brain [32].

Here, we demonstrate that Rab32 and Rab38, but no other GTPases tested, directly interact with LRRK2. In yeast two-hybrid experiments we identify a predicted coiled-coil motif containing region within the aminoterminus of LRRK2 as a possible interacting domain. While microscopy analyses display a co-localization of Rab32 and LRRK2 at pericentrosomal recycling endosomes in addition to transport vesicles, overexpression of a constitutively active mutant of Rab32 leads to an increased co-localization at Rab7 and Rab9 positive perinuclear late endosomes/MVBs. Subcellular fractionation reveals that overexpression of constitutively active Rab32 decreases the amount of LRRK2 in mitochondria and lysosome containing fractions. Rab32-dependent localization of sub-cellular LRRK2 distribution thereby demonstrates a role for Rab32 in LRRK2 sorting and transport.

## Material and Methods

### Plasmids and cloning

Human Rab32 was amplified from pEGFP-Rab32 wt vector, which was a kind gift from C. Wasmeier from M. Seabra's group, by PCR (primer forward: AGAATTCCATATGGCGGGCGGAGGAGCC; primer reverse: TACCTAGGTCAGCAACACTGGGATTTGTTC) and cloned into the plasmid pAS2-1 to construct the yeast two-hybrid bait plasmid pAS-Rab32 wt [17]. The same primers were used to clone the constitutively active GTP bound Rab32 Q85L mutant in the pAS2-1 vector, using pDsRed-Monomer-Rab32 Q85L as template. This construct was generated by site directed mutagenesis from pDsRed-Monomer-Rab32 wt using the following primers: forward: GGGACATCGCGGGGCTGGAGCGATTTGGCAAC; reverse: GTTGCCAAATCGCTCCAGCCCGATGTCCC. pDsRed-Monomer-Rab32 wt was constructed similar to pAS-Rab32 (primer forward: AAGAATTCTATGGCGGGCGGAGGAGC; primer reverse: GTGGATCCTCAGCAACACTG). The yeast two-hybrid plasmids pAS-Rab38 wt and pAS-Rab38 Q69L were cloned by PCR (primer forward: CAGAATTCCATATGCAGACACCTCACAAG; primer reverse: CTGGATCCCCTAGGATTTGGCACAGCC) amplifying murine Rab38 sequences from pEF-FLAG-Rab38 wt and pEF-FLAG-Rab38 Q69L, which were a kind gift from M. Fukuda, Tohoku University, Sendai, Japan [18]. Other vectors used in this study were constructed by sub-cloning: pACT2-LRRK2, pACT2-LRRK2-C, pACT2-LRRK2 1-266, pACT2-LRRK2 265-552, pEGFP-Rab32 Q85L, pGEX42-Rab32 wt, pECFP-Rab32 wt and -Q85L and pEGFP-Rab32 Q85L. All vectors constructed by PCR were verified by sequencing. The pcDNA3-LRRK2-EGFP plasmid was described before [3]. The plasmids pEYFP-Endo (RhoB) and pEGFP-Rab5A were a kind gift from Theresia Stradals group, University Muenster, Germany. pEGFP-Rab11B wt was a gift from Beate Schlierf, University Erlangen, Germany [33].

### Antibodies

Antibodies used in Western blotting were anti-human Rab32 and anti-LC3B antibodies from Sigma (St. Louis, MO, USA) and used at a 1∶1000 dilution. Mouse anti GAPDH was diluted 1∶5000 (Invitrogen, Carlsbad, USA). The rat anti-LRRK2 (1E11) hybridoma supernatant was produced by E. Kremmer, Helmholtz Zentrum München, Germany and the antibody was applied at a 1∶50 dilution [34]. For the analysis of the GFP-Trap experiments we used the MJFF2 rabbit anti LRRK2 antibody (Abcam, Cambridge, UK) at a 1∶2000 dilution and a mouse anti GFP (JL-8, Clontech, Mountain View, USA) antibody at a dilution of 1∶4000. HRP-coupled antibodies were used as secondary antibodies for Western blotting. Anti-rabbit and anti-mouse secondary antibodies were obtained from Cell Signaling Technology, Danvers, MA, USA, and diluted 1∶1000. Anti-rat IgG HRP was from Jackson Immunoresearch, Newmarket, UK, and diluted 1∶5000.

For immunofluorescent stainings anti-Rab7 and -Rab9 antibodies (Cell Signaling Technology, Danvers, MA, USA) were used at dilutions of 1∶50 and 1∶100, respectively. The mouse anti-Rab11 (clone47) antibody was obtained from Transduction Laboratories, Lexington, USA). Mouse anti-β-tubulin hybridoma supernatant (clone E7; Developmental Studies Hybridoma Bank, University of Iowa, USA) was a kind gift from Sven Bogdan, University Muenster, Germany. Both were used at 1∶50 dilutions. Supernatants from mouse hybridoma to detect Rab6A (5B10) and Rab1B (1E7) were described earlier [35], [36], [37]. Secondary antibodies were coupled to Oyster594 (Luminartis GmbH, Muenster, Germany), cy3 or Alexa488 (Jackson Immunoresearch, Newmarket, UK). U. Schulze kindly provided us with the mouse anti-LAMP2 antibody (clone H4B4; Developmental Studies Hybridoma Bank, University of Iowa, USA), dilution was 1∶250 in secondary immunofluorescence and 1∶5000 in Western blots.

### Yeast two-hybrid

The human lung Matchmaker cDNA Library (Clontech, Mountain View, CA, USA) was a kind gift from Stefan Ludwigs Lab, UKM, Muenster, Germany. The reporter yeast strain Y190 (Clontech, Heidelberg, Germany) was co-transformed and colonies were analyzed as described previously [38], [39]. The reporter strain Gold (Clontech, Heidelberg, Germany) was co-transformed similar to the Y190 strain according to the manufacturer's instructions.

### GST-pulldown and immunoprecipitation

GST-fusion proteins of Rab32 and GST alone as control were expressed from pGEX-vectors in *E. coli* BL21 for three hours at room temperature after induction with 1 mM IPTG and lysed by sonication in PBS pH 7.4 containing EDTA-free protease inhibitor cocktail *complete* (Roche Diagnostics, Mannheim, Germany). After addition of Triton X-100 (final concentration 1%) followed by 30 minutes incubation on ice, the lysates were cleared by centrifugation. GST fusion proteins were purified using 10 µl bed volume Glutathion Sepharose 4B (GE Healthcare Bioscience, Freiburg, Germany). 5 µg of each GST fusion protein and GST as negative control were used for each experiment. After loading the beads and subsequent washing, NIH3T3 cell lysates were added and incubated overnight at 4°C in an overhead shaker. Cell lysates were made by adding *pulldown* buffer (10 mM Tris pH 7.4, 150 mM NaCl, 1 mM CaCl_2_, 1 mM MgCl_2_, 0,2% Triton X-100, *complete EDTA free*) to the three times PBS washed cell culture dish. Subsequently, cells were harvested with a cell scraper and the lysate was cleared by 1 hour centrifugation at 23,000× g, 4°C. After 24 hours of incubation beads were washed in 200 µl *pulldown* buffer three times and subjected to Western blot analysis. Alternatively, Glutathione HiCap matrix (Qiagen, Hilden, Germany) was used according to the manufacturer's instructions.

For co-immunoprecipitation experiments confluently grown cells on a 15 cm cell culture dish were harvested in co-IP buffer (50 mM Tris pH 7.4, 150 mM NaCl, 1.5 mM MgCl, 4 mM EDTA, 10% glycerol, 1% Triton X-100, *complete* EDTA-free). After clearing the lysates by centrifugation, 1–2 µg of the antibody was added. After 24 h incubation at 4°C in a rotator 10 µl of Protein G Sepharose 4 Fast Flow (GE Healthcare Bioscience, Freiburg, Germany) beads were added followed by incubation for another hour. Thereafter, the beads were washed with 200 µl PBS containing 0.2% Triton X-100 three times and the samples were subjected to Western blot analysis.

For co-immunoprecipitation using the GFP-Trap kit (Chromotek, Planegg-Martinsried, Germany) cells were harvested in a modified GFP-Trap buffer (10 mM Tris pH 7.5, 150 mM NaCl, 0.5 mM EGTA, 0.5% NP40, 200 µM sodium orthovanadate, *complete* EDTA free). The further procedure was according to the manufacturers instructions.

### Western blot

For Western blotting, cell lysates were mixed with 4× loading dye (40% (v/v) glycerol, 8% (w/v) SDS, 32% (v/v) 1 M Tris/HCl pH 6.8, 0.04% (v/v) bromophenol blue and 20% β-mercaptoethanol) and incubated 5 min at 95°C. For LRRK2 samples incubation time was reduced to 60–90 seconds. Samples were subjected to SDS-PAGE [40]. Proteins were immobilized on PVDF by semi dry Western blotting. The membrane was blocked in 5% skimmed milk powder in PBS-T (0.1% Tween20 in PBS) for 1 hour at RT or over night at 4°C. Primary antibodies were diluted in blocking solution and incubated for 60 min at RT or over night at 4°C for the anti-LRRK2 antibodies. The secondary antibodies (HRP conjugated) were diluted in blocking solution and incubated for 30 min at RT. Subsequently, the membrane was washed in PBS-T 6× for at least 5 min. Detection of proteins was carried out on X-ray films using chemiluminescence.

### Cell culture and transfection

NIH3T3 (ATTC number: CRL-1658) cells were cultured in DMEM supplemented with 10% FBS and 4 mM glutamine. One day prior to transfection, 5×10^5^ NIH3T3 cells were seeded in a 3.5 cm cell culture dish containing up to three 12 mm cover slides. Co-transfection of either pDsRed-Monomer-Rab32 wt or -Q85L and pcDNA3-LRRK2-EGFP was carried out using PolyFect (Qiagen, Hilden, Germany) or Lipofectamine 2000 (Invitrogen, Karlsruhe, Germany) according to the manufacturer's instructions. All other transfections were performed by using TurboFect (Thermo Scientific, Schwerte, Germany) according to the manufacturer's instructions.

The renal proximal tubule cell line IHKE-1 was a kind gift from Thomas Weide, UKM, Muenster. Cells were grown in DMEM/Ham's F12 supplemented with 1% FBS, 15 mM HEPES (pH 7.2), 44 mM NaHCO_3_, 1 mM sodiumpyruvate, 4.5 mM L-glutamine, 36 ng/ml hydrocortisol, 10 ng/ml EGF, 5 µg/ml insulin, 5 µg/ml transferrin and 5 ng/ml sodium selenite.

Like NIH3T3 cells, IHKE-1 cells were transfected with TurboFect during passaging. 1×10^5^ cells were mixed with the TurboFect-DNA dilution and plated in a 48 well cell culture dish. The next day, cells were transferred to a 3.5 cm cell culture dish containing 12 mm cover slides or two 3.5 cm dishes were transferred to one 6 cm cell culture dish. After 24 hours cells were either fixed or harvested for further experiments. To generate IHKE-1 cell lines stably expressing GFP-Rab32 wt or GFP-Rab32 Q85L, cells were transfected as described above and then incubated with G418. After a few weeks colonies from surviving cells were treated with trypsin, subjected to 48 well plates and grown to confluence. The best of the resulting clones were further cultivated and used for this study.

### Secondary immunofluorescence

For secondary immunofluorescence cells were washed three times with ice cold PBS and were fixed for 10 min on ice with 4% PFA in 250 mM HEPES, pH 7.4. An additional incubation step for 30 min at room temperature (RT) followed. After washing with PBS three times cells were incubated with 50 mM NH_4_Cl for 5 min at RT. The cells were washed three times with PBS and permeabilized with 0.2% Triton X-100 in PBS for 5 min. Thereafter, cells were washed three times with PBS containing 0.2% gelatine. Unspecific antibody binding sites were blocked with 10% normal goat serum in PBS+0.2% gelatine for 30 min followed by incubation with the primary antibody in PBS+0.2% gelatine for 60 min. After that the cells were washed three times with PBS+0.2% gelatine and subsequently incubated with secondary antibody in PBS+0.2% gelatine for 20 min. Before embedding with Mowiol (Sigma Aldrich Chemie GmbH, Taufkirchen, Germany) supplemented with DAPI and DABCO, the cells were washed three times with PBS and three times with ddH_2_O. The protocol was carried out in drops on Parafilm beginning from the neutralization step. The samples were analyzed with a Leitz Diaplan Fluorescence microscope (Leica, Darmstadt, Germany) equipped with PL Fluotar 100× - 1.32 NA and PL Fluotar 50× - 1.00 NA objectives or a Zeiss LSM5 live (Carl Zeiss, Jena, Germany) with a Plan Apochromat 63× – 1.40 NA objective.

### Live cell imaging

For live cell imaging cells were grown in 8 well microscopic slides (IBIDI, Martinsried, Germany) and transfected with Lipofectamine 2000 (Invitrogen, Karlsruhe, Germany) according to the manufacturer's instructions. Cells were analyzed on an LSM5 live microscope (Carl Zeiss, Jena, Germany). All images were taken in the *live* mode as 8 bit images using a two-track recording setup. Green and red channels were recorded in parallel for each time point. Laser power and detector gain were adjusted as needed.

### Microscopic imaging and determination of endosomal size

Images were taken with a Leitz Diaplan Fluorescence microscope equipped with PL Fluotar 100× - 1.32 NA and PL Fluotar 50× - 1.00 NA objectives. Images were taken with an Olympus XM10 camera as 16 bit multipage.tif files, filters for green (excitation: 450–490 nm, emission: 515–560 nm) and red (excitation: 540–580 nm, emission: 607–682 nm) fluorescence guaranteed no bleed trough. Images were taken with constant exposure time and analyzed with ImageJ [41]. For quantifying the size of perinuclear late endosomes/MVBs the channel showing Rab7 was blurred with a 2 px median filter followed by subsequent background subtraction with a rolling ball radius of 20 px. Thereafter, an adequate threshold was set manually and the image was binarized. Data for analysis were generated by selecting the perinuclear areas with the wand tool and measuring their sizes.

### Subcellular fractionation

For subcellular fractionation IHKE-1 or IHKE-1 cells stably transfected with either GFP-Rab32 wt or GFP-Rab32 Q85L were grown on 15 cm diameter cell culture dishes to confluence. Then, cells were washed 3 times with PBS, 4°C, and cell culture plates were frozen at −70°C. To disrupt the cells we added 450 µl fractionation buffer (250 mM sucrose, 20 mM HEPES (pH 7.4), 10 mM potassium chloride, 1.5 mM magnesium chloride, 1 mM EDTA, 1 mM EGTA, 1 mM DTT, *complete* EDTA-free (Roche Diagnostics, Mannheim, Germany)) and immediately scraped the cells off. The lysate was collected to a tube and snap frozen in liquid nitrogen and thawed 5 times. The resulting lysate was pressed through a 27 Gauge needle with a syringe 20 times. Appropriate disruption of the cells was assessed by light microscopy. This whole cell lysate was separated into the different fractions by subsequent centrifugation steps. First, centrifugation at 720× g for 5 minutes was for pelleting nuclei and bigger cell debris. The resulting supernatant then was centrifuged at 20,000× g for 20 minutes to sediment high density organelles like lysosomes and mitochondria. The pellet was resuspended in 150 µl fractionation buffer supplemented with 0.1% SDS. The resulting lysate was used as crude mitochondria and lysosome containing fraction (CLM). The supernatant containing light membrane fragments and cytosolic proteins was used as C/M fraction.

### Statistical analysis

Statistical analysis was performed by two-sided Student's T-test in Excel (Microsoft Corporation, Redmond, WA, USA) assuming normal distribution. Differences with p-values less than 0.05 were considered as significant.

## Results

### Yeast two-hybrid Interactions between LRRK2 and Rab32

**Figure 1:**
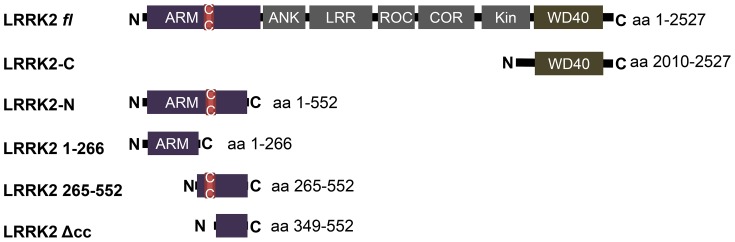
LRRK2 constructs used in this study. ARM: armadillo repeats; ANK: ankyrin repeats; LRR: leucine rich repeats; ROC: Ras of complex; COR: C-terminal of ROC; Kin: kinase domain; WD40: WD40 domain; c/c: coiled coil motif (aa 319–348).

Several studies have described the role of Rab32 in the context of melanogenesis together with the most closely related Rab38 [17], [24]. Both GTPases were described to act mostly in a redundant fashion in melanogenic transport pathways. However, Rab32 is not only expressed in melanin containing cells - several groups have demonstrated by Northern as well as Western blot analyses that lung tissue contains high levels of Rab32 mRNA and protein [27], [42], [43]. To obtain further insight into the role of Rab32 in non-melanogenic cells, we screened a human lung library in a yeast two-hybrid assay using Rab32 *wildtype* (wt) as bait. We screened up to 3.7×10^7^ clones and isolated four clones which caused a strong activation of reporter genes in the presence of Gal4BD-Rab32 wt fusion proteins. Sequence analyses identified these clones as a DNA fragment encoding the amino acids 1–552 of human *leucine rich repeat kinase* 2 (LRRK2, Figure 1).

### GST-Rab32 pulldowns and immunoprecipitation of endogenous LRRK2

**Figure 2:**
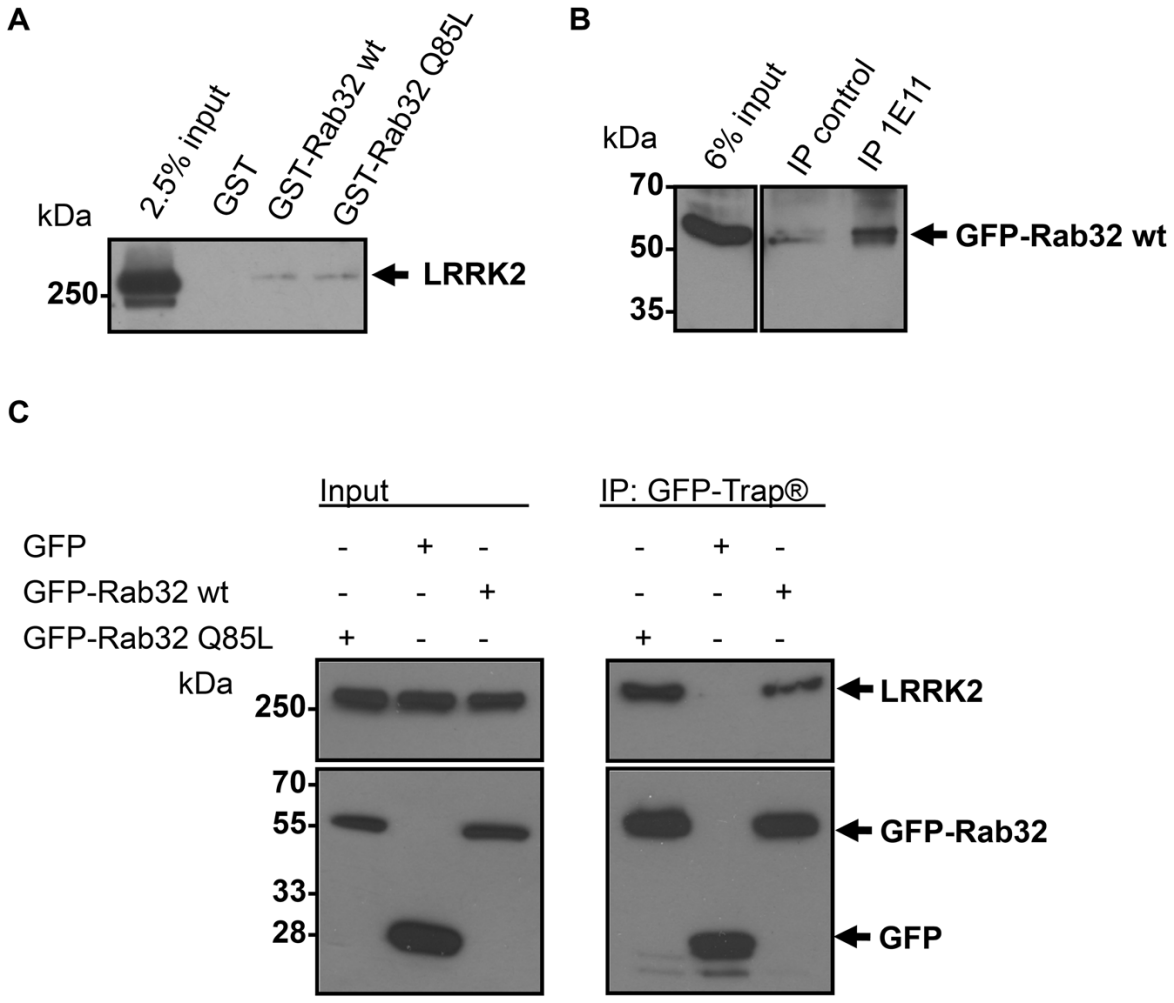
Binding of endogenous LRRK2 by the small GTPase Rab32. (A) GST-Rab32 wt, GST-Rab32 Q85L or GST as control was applied to glutathione agarose beads followed by incubation with NIH3T3 lysate overnight. Samples were analyzed by 6% SDS-PAGE and subsequent Western blot analysis to detect LRRK2. n≥3 independent experiments. (B) Lysates from IHKE-1 cells stably expressing GFP-Rab32 wt were incubated overnight with an anti-LRRK2 antibody (1E11). IP control = no antibody was added. Co-precipitated GFP-Rab32 wt was detected using an anti-Rab32 antibody. n = 3 independent experiments. (C) Lysates from IHKE-1 cells expressing GFP-Rab32 wt, GFP-Rab32 Q85L or GFP as control were subjected to immunoprecipitation by the GFP-Trap kit. Co-precipitated endogenous LRRK2 was detected using an anti LRRK2 antibody. n = 2 independent experiments.

Although yeast two-hybrid experiments are suitable for detecting new interacting partners of proteins, this method could identify false positive results and therefore the interaction between Rab32 and LRRK2 required confirmation by independent methods. Bacterially expressed GST-Rab32 wt and GST-Rab32 Q85L was coupled to glutathione agarose beads and incubated with lysates from NIH3T3 cells. These cells show highest endogenous LRRK2 expression of all cell lines we have tested. We were able to pull down endogenous LRRK2 from NIH3T3 cell lysates using GST fusion proteins of Rab32, but not with GST-Rab1A (not shown) or GST alone (Figure 2A).

To further confirm the direct interaction of LRRK2 and Rab32, co-immunoprecipitation experiments were performed. We generated an IHKE-1 cell line stably expressing GFP-Rab32 wt (IHKE GFP-Rab32 wt) and GFP-Rab32 Q85L (IHKE GFP-Rab32 Q85L). This human renal cell line was made from epithelial cells of the proximal tubule, which was demonstrated to have high LRRK2 abundance [14], [44], [45]. IHKE-1 cells have the advantage of relatively high LRRK2 expression and were easy to transfect. Furthermore, Rab32 is abundant in kidney cells [27], [42], [43]. Figure S1 shows the expression of endogenous Rab32 in untransfected IHKE-1 cells and IHKE GFP-Rab32 wt cell lines and GFP-Rab32 wt in the stably transfected cell line (Figure S1). We pulled down endogenous LRRK2 using the 1E11 anti-LRRK2 antibody and probed for GFP-Rab32 wt by Western blot analysis. We were able to detect GFP-Rab32 wt following immuno-precipitation of LRRK2 (Figure 2B). In contrast, there was a minor background in the absence of antibody (Figure 2B) or a control antibody (data not shown). Unfortunately, we could not detect endogenous Rab32 due to strong non-specific signals at the molecular weight of the small GTPase.

Due to the lack of available antibodies suitable for the immunoprecipitation of endogenous Rab32 we used the GFP-Trap system as an alternative approach to confirm its interaction with LRRK2. Recombinantly expressed GFP-Rab32 wt or GFP-Rab32 Q85L allowed the co-precipitation of endogenous LRRK2 from IHKE-1 cell lines while no LRRK2 bound to a GFP-only control (Figure 2C).

### Rab32 specificity of the LRRK2 interaction

Generally, small GTPases are known to bind their effectors in a highly specific manner. On the other hand, many interacting partners are recognized by different Rabs at distinct or overlapping binding sites. Notably, LRRK2 is known to bind several small GTPases [8], [9], [10], [15], [16]. Thus, further investigations were needed to test, whether the interaction is specific for Rab32. We used an assortment of Rab cDNA molecules as bait constructs in yeast co-transformation assays (Table 1). LRRK2-N induced cell growth and α-galactosidase activity only when co-transformed with Rab32 or the closely related Rab38 plasmids. Other GTPases tested did not interact, as evidenced by the inability to rescue growth and α-galactosidase activity. Furthermore, we used co-transformation of LRRK2-N with Tau as a negative control. Tau is a microtubule binding protein forming neurofibrillary tangles in Alzheimer's disease and is also involved in Parkinson's disease. However, there are no apparent direct interactions between Tau and LRRK2 [46], [47], [48], [49].

**Table 1:** Rab binding specificity of LRRK2-N.

bait plasmid	prey plasmid	QDO+X-α-gal+aureobasidin A
pAS2-1-**Rab32** wt	pACT2-**LRRK2-N**	**+**
pAS2-1-**Rab38** wt		**+**
pAS2-1-**Rab1B** Q67L		**−**
pAS2-1-**Rab1B** Q67R		**−**
pAS2-1-**Rab11A** wt		**−**
pAS2-1-**Rab5A** wt		**−**
pAS2-1-**Rab6A** Q72L		**−**
pAS2-1-**Rab6A** Q72R		**−**
pAS2-1-**Rab7**ΔC		**−**
pAS2-1-**tau**		**−**

After co-transformation of the yeast strain Gold with the indicated plasmids, cells were grown on synthetic media lacking adenine and histidine supplemented with 125 ng/ml aureobasidin and 40 µg/ml X-α-gal. Cell growth and blue color indicated that the proteins interact.

− no or reduced growth on selection media and no blue color, + colony growth on selection media and blue color. n≥3 independent experiments.

### Mapping the Rab32 binding domain of LRRK2

To investigate which part of the LRRK2 protein acts as Rab32 binding domain, we generated several deletion mutants of LRRK2 and tested them in yeast two-hybrid co-transformation assays (Figure 1, Table 2). A clear interaction signal was observed with the LRRK2-N deletion mutant and the truncated LRRK2-N clone, LRRK2 265–552 (Table 2). No interaction was observed with the deletion mutants LRRK2 1–266 and LRRK2-C indicating that the Rab32 binding domain lies within the amino acids (aa) 267 to 552. The LRRK2 full length construct also did not give a clear positive signal in these experiments, although we could demonstrate the interaction of endogenous LRRK2 with Rab32, as shown before (Figure 2). It is possible, that issues regarding protein folding or posttranslational modifications in the yeast cells lead to a false negative result in this experiment.

**Table 2:** Mapping of Rab32 binding domain within LRRK2.

bait vector	prey vector	QDO+X-α-gal+aureobasidin A
pAS2-1 **Rab32 Q85L**	pACT2-**LRRK2**	**−**
	pACT2-**LRRK2-N**	**+**
	pACT2-**LRRK2 1-266**	**−**
	pACT2-**LRRK2 265-552**	**+**
	pACT2-**LRRK2-C**	**−**

The yeast strain Gold was co-transformed with the indicated plasmids. Cells were grown on synthetic media lacking adenine and histidine. The medium was supplemented with 125 ng/ml aureobasidin and 40 µg/ml X-α-gal. Cell growth and blue color indicated that the proteins interact.

− no or reduced growth on selection media and no blue color, + colony growth on selection media and blue color. n≥3 independent experiments.

Very little is known about the function of the LRRK2 aminoterminus, which displays armadillo repeat structures. The smallest Rab32 binding motif we found in our screen contained a hypothetical coiled-coil motif spanning the amino acids 319 to 348. We found this motif by entering the first 552 residues of LRRK2 to the coils algorithm (Figure S2) [50]. To elucidate, whether this motif serves as the Rab32 interacting domain we generated an additional LRRK2 deletion mutant that lacks this hypothetical coiled-coil motif (LRRK2 aa 349–552: LRRK2Δcc, Figure 1). Yeast co-transformation assays revealed that only the aa 265–552 fragment binds to Rab32, but not the one lacking the region with the predicted coiled-coil motif (Table 3). Therefore, we concluded that Rab32 is interacting with LRRK2 via this coiled-coil motif.

**Table 3:** Mapping of Rab32 binding motif within LRRK2 aminoterminus.

bait vector	prey vector	β-gal
pAS2-1 **Rab32 Q85L**	pACT2-**LRRK2 265–552**	**+**
	pACT2-**LRRK2 Δcc**	**−**

After co-transformation of the yeast strain Y190 with the indicated plasmids with and without the hypothetical coiled-coil motif, cells were grown on synthetic media lacking histidine, supplemented with 30 mM 3 AT. Cell growth on these media and blue staining in subsequent β-gal-filter assays indicated direct interaction of the proteins.

no growth on selection media or staining in ß-galactosidase filter assay, + growth on selection media and blue staining in in ß-galactosidase filter assay. n = 3 independent experiments.

### Analysis of Rab32 and LRRK2 co-localization in various intracellular compartments

**Figure 3:**
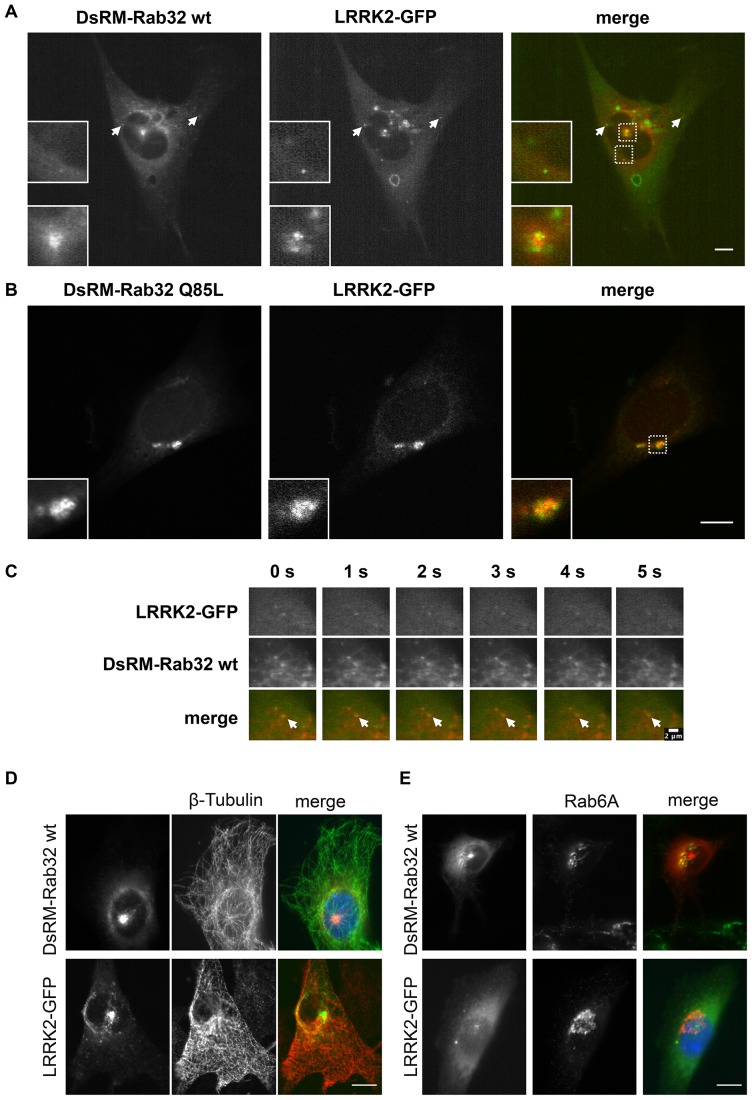
Co-localization analysis of LRRK2-GFP and DsRed-Monomer-Rab32. (A) NIH3T3 cells were co-transfected with plasmids encoding DsRed-Monomer-Rab32 wt and LRRK2-GFP. Living cells were imaged using a Zeiss LSM5 live microscope. The image shows a still frame from Movie S1. Scale bar = 10 µm. (B) NIH3T3 cells were co-transfected with plasmids encoding DsRed-Monomer-Rab32 Q85L and LRRK2-GFP. After 48 hours the cells were fixed in 4% PFA and subsequently analyzed with a laser scanning microscope. Scale bar = 10 µm. (C) Co-transport and sorting of LRRK2-GFP by DsRed-Monomer-Rab32 wt. Image series are a detail view from Movie S2. Both channels were recorded simultaneously every second. Scale bar = 2 µm. (D and E) NIH3T3 cells expressing either DsRed-Monomer-Rab32 wt or LRRK2-GFP were fixed and subsequently subjected to immunofluorescence labeling of β-tubulin (D) or the Golgi marker Rab6A (E). Scale bar = 10 µm.

Following verification and mapping of the Rab32-LRRK2 interactions, we analyzed their intracellular co-localization. NIH3T3 cells were co-transfected with plasmids encoding DsRed-Monomer-Rab32 wt and LRRK2-GFP. After 48 hours cells were analyzed by live cell imaging. DsRed-Monomer-Rab32 and LRRK2-GFP partially co-localized in a structure at the center of the cell (Figure 3A, Movie S1). Perinuclear as well as peripheral punctate co-localization was also visible (Figure 3A, arrows).

In many cases the constitutively active mutant of a small Rab GTPase looks like an exaggerated version of its wildtype and thereby more explicit in phenotype. This has been observed, for example, for Rab5 or Rab7, which form giant early endosomes or perinuclear aggregates of late endosomes/MVBs, respectively [51], [52], [53]. To investigate LRRK2-GFP co-localization with constitutively active Rab32 we co-transfected plasmids encoding DsRed-Monomer-Rab32 Q85L and LRRK2-GFP in NIH3T3 cells and compared them with DsRed-Monomer Rab32 wt co-expressing cells. Surprisingly, the Q85L mutant looks rather different from the wildtype (Figure 3B). It had a more diffuse appearance, but most characteristic for the constitutively active mutant are large perinuclear aggregates that occurred in over 90% of all Rab32 Q85L expressing NIH3T3 cells we analyzed. We observed significant co-localization of DsRed-Monomer-Rab32 Q85L and LRRK2-GFP in these perinuclear aggregates.

Live cell imaging revealed co-transport and sorting events of LRRK2-GFP and Ds-Red-Monomer-Rab32 wt at vesicles (Figure 3C, Movies S1 and S2). It was obvious that the co-transport was not only directed to the center of the cell, but also away from it (Movie S1). This indicates a presumable role for Rab32 in LRRK2 sorting and transport in the cell.

To study the co-localization of Rab32 and LRRK2 in more detail, we transfected cells with plasmids encoding either DsRed-Monomer-Rab32 wt or LRRK2-GFP. Immunofluorescence staining for β-tubulin showed that the area of co-localization occurred pericentrosomal at the microtubule organizing center (MtOC, Figure 3D). Both Rab32 and LRRK2 were also reported to localize to the Golgi apparatus [3], [14], [15], [29]. To examine, whether the Golgi apparatus is the compartment of co-localization, cells were either transfected with plasmids encoding DsRed-Monomer-Rab32 wt or LRRK2-GFP and stained with an antibody for the Golgi marker protein Rab6A (Figure 3E). We could demonstrate co-localization of DsRed-Monomer-Rab32 wt and Rab6A, but no co-localization of Rab6A and LRRK2-GFP was detectable. Therefore we concluded that the compartment of the interaction is not the Golgi apparatus. The perinuclear aggregates that we observed in the constitutively activated mutant co-localize neither with β-tubulin nor with Rab6A (data not shown).

### Localization of Rab32 with endosomal marker proteins

**Figure 4:**
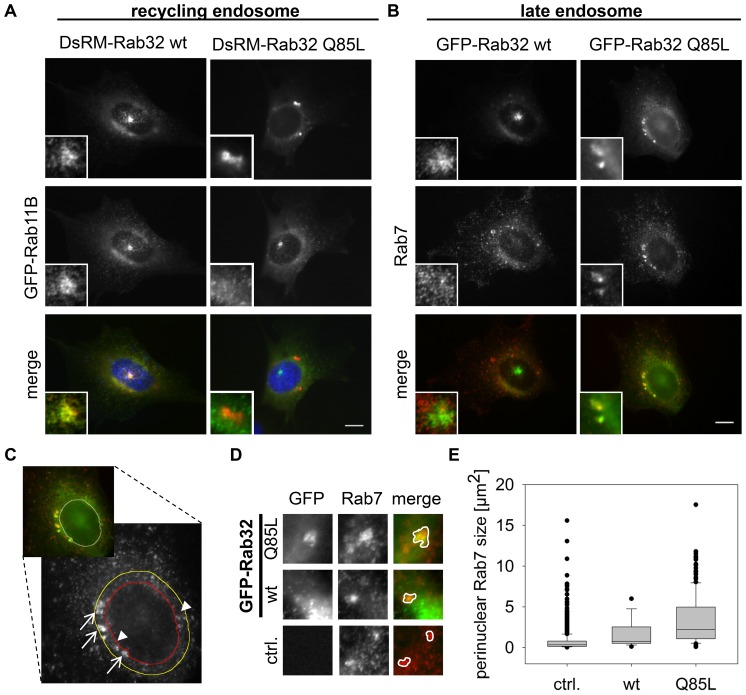
Co-localization analysis of Rab32 wt and the constitutively active mutant Rab32 Q85L with different endosomal markers. (A) NIH3T3 cells were co-transfected with plasmids encoding the recycling endosome marker GFP-Rab11B and DsRed-Monomer-Rab32 wt or DsRed-Monomer-Rab32 Q85L, fixed and analyzed by fluorescence microscopy. Scale bar = 10 µm. (B) Cells were transfected with plasmids encoding for GFP-Rab32 wt or GFP-Rab32 Q85L followed by fixation and subsequent immunofluorescence staining of Rab7. Scale bar = 10 µm. (C–E) NIH3T3 cells expressing either GFP-Rab32 wt or GFP-Rab32 Q85L were fixed and stained for Rab7. (C) Microscopic analysis of GFP-Rab32 Q85L that co-localized with endogenous Rab7 (arrows) in the perinuclear area. Non co-localizing Rab7 was indicated by arrowheads. The perinuclear area was defined by the red (outlines nucleus) and the yellow line (outer border for perinuclear area). The image illustrates the cellular area used for the following analysis. (D) Rab7 perinuclear aggregates co-localizing with GFP-Rab32 wt or GFP-Rab32 Q85L and non co-localizing ones. ctrl. = control (untransfected IHKE-1 cells) (E) Quantification of perinuclear Rab7-positive structures. ctrl. (control): perinuclear Rab7 in untransfected cells; wt/Q85L: perinuclear Rab7 co-localizing with GFP-Rab32 constructs. control/Rab32 wt/Rab32 Q85L: n = 1660/18/167 structures in 67/18/60 cells, 1/1/3 independent experiments. Statistical significance was tested by a Student's T-test: p_control-wt_ = 0.02; p_control-Q85L_<0.005; p_wt-Q85L_ = 0.02. Scale bar = 10 µm.

Rab32 is known to organize lysosome related organelles (LRO) and therefore to act within the endosomal system [17], [24]. We determined to which endosomal system Rab32 wt and constitutively active Rab32 Q85L localize by co-transfecting the corresponding constructs with plasmids encoding GFP-Rab11B (recycling endosomes) or GFP-Rab5A (early endosomes). We could not detect co-localization between DsRed-Monomer-Rab32 wt or -Q85L and GFP-Rab5A at early endosomes (Figure S3A). This is in good agreement with the results from other groups, who examined the role of Rab32 in the context of melanogenesis [25]. In contrast to Rab5A we observed substantial co-localization of GFP-Rab11B with DsRed-Monomer-Rab32 wt at the pericentriolar region (Figure 4A) indicating the recycling endosome nature of this structure. Compared to DsRed-Monomer-Rab32 wt, DsRed-Monomer-Rab32 Q85L displayed no co-localization with GFP-Rab11B, especially not at the perinuclear aggregates. For the detection of late endosomes/MVBs we transfected cells with either GFP-Rab32 wt or -Q85L constructs. After 24 hours cells were fixed and stained using an anti-Rab7 antibody (Figure 4B). In contrast to the results obtained for GFP-Rab11B, Rab7 strongly co-localized with the perinuclear aggregates of DsRed-Monomer-Rab32 Q85L, but not with the pericentrosomal GFP-Rab32 wt. In NIH3T3 cells expressing just LRRK2-GFP we were able to detect a partial co-localization with Rab7 and Rab11 (Figure S4). Similar to the Rab7 experiment did co-staining of GFP-Rab32 wt or -Q85L and Rab9. This small GTPase is known for its function in mannose-6-phosphate receptor recycling from late endosomes to the TGN [54]. We also observed only limited co-localization of GFP-Rab32 wt and Rab9, but substantial co-localization at the perinuclear aggregates of GFP-Rab32 Q85L transfected cells (Figure S3B). Here, typically the brightest Rab9 intensity is next to the Rab32 signal, but lower intensity areas matched with the aggregates. Although Rab32 fused to fluorescent proteins (GFP, DsRed-Monomer) has a very “typical” phenotype in NIH3T3 cells, we found similar distribution and co-localization of Rab32 with the markers (as far as examined) in different cell types like IHKE-1 (Figure S5 and S6A), HeLa or A549 cells (data not shown). In conclusion, these data support a role of Rab32 in transport processes from the Golgi apparatus via recycling endosomes to late endosomes/MVBs or lysosomes.

### Effects of wildtype and constitutively activated Rab32 on late endosomes

It can easily be concluded from Figure 4B that constitutive activation of Rab32 leads to an altered perinuclear accumulation of Rab7 (Figure 4B). Therefore we measured the area of Rab7 staining that co-localized with GFP-Rab32 wt or -Q85L (Figure 4C, arrows) and additionally the Rab7 that did not co-localize with them in a small perinuclear region as indicated in Figure 4C (Figure 4 C, arrowheads). Figure 4D shows that this perinuclear Rab7 formed larger aggregates upon GFP-Rab32 wt and especially -Q85L overexpression (Figure 4D). For perinuclear Rab7 structures that co-localize with GFP-Rab32 wt we determined a median size of 0.77 µm^2^ and for GFP-Rab32 Q85L co-localizing with Rab7 2.22 µm^2^ (Figure 4E). However, non co-localizing Rab7 in the perinuclear region in untransfected control cells had an area of just 0.33 µm^2^. Statistical analysis revealed that these differences were highly significant (p<0.005). Rab7 positives areas in the perinuclear region of transfected cells, where no co-localization with Rab32 was observed, were about the size of the untransfected control (GFP-Rab32 wt: 0.40 µm^2^; GFP-Rab32 Q85L: 0.34 µm^2^). This indicated a Rab32 activity-dependent effect on the cellular localization of Rab7, which is a major regulator of late endosome maturation and lysosome fusion [55].

To test, whether the changes in the degree of GFP-Rab32 wt or -Q85L and Rab7 co-localization in NIH3T3 cells is a general feature of Rab32 behavior, we repeated the co-localization experiments with the Rab32 constructs and endogenous Rab7 in a human cell line, the IHKE-1 cells. We cultivated IHKE-1 cells stably transfected with GFP-Rab32 wt or -Q85L (IHKE GFP-Rab32 wt or IHKE GFP-Rab32 Q85L) on cover slips, fixed them and subsequently stained for Rab7 localization by secondary immunofluorescence. We could demonstrate that Rab7 exhibits a significantly higher overlap with GFP-Rab32 Q85L than GFP-Rab32 wt does (Figure S6A). In contrast, LAMP2 shows co-localization with both GFP-Rab32 wt and GFP-Rab32 Q85L, but increased co-localization with the latter was not obvious (Figure S6B). LAMP2 is a typical marker of lysosomes but also abundant in late endosomes/MVBs and was shown to co-localize not only with Rab7 but also with Rab32 wt and -Q85L before [29], [56]. From our observations, we conclude that Rab32 targets its interacting partners to late endosomes/MVBs. Activated Rab32 stimulates the formation of enlarged perinuclear late endosomes/MVBs. Therefore it seems very interesting to investigate, whether the novel Rab32 interacting protein LRRK2 changes its more indistinct cellular distribution pattern upon activation of the Rab32 GTPase and is targeted by activated Rab32 to late endosomes/MVBs via recycling endosomes.

### Rab32 mediates effects on the intracellular localization of LRRK2

When we analyzed the localization of Rab32 in NIH3T3 cells, we found considerable differences between the Rab32 wildtype and the permanently GTP bound Rab32 Q85L mutant (Figure 3A, B and Figure 4). In fact, Rab32 wt displays the major degree of co-localization with LRRK2-GFP at pericentriolar recycling endosomes and transport vesicles, while the constitutively active mutant co-localizes with LRRK2-GFP in perinuclear aggregates positive for the late endosomal marker Rab7. From this observation we hypothesized that Rab32 influences LRRK2 localization inside the cell.

**Figure 5:**
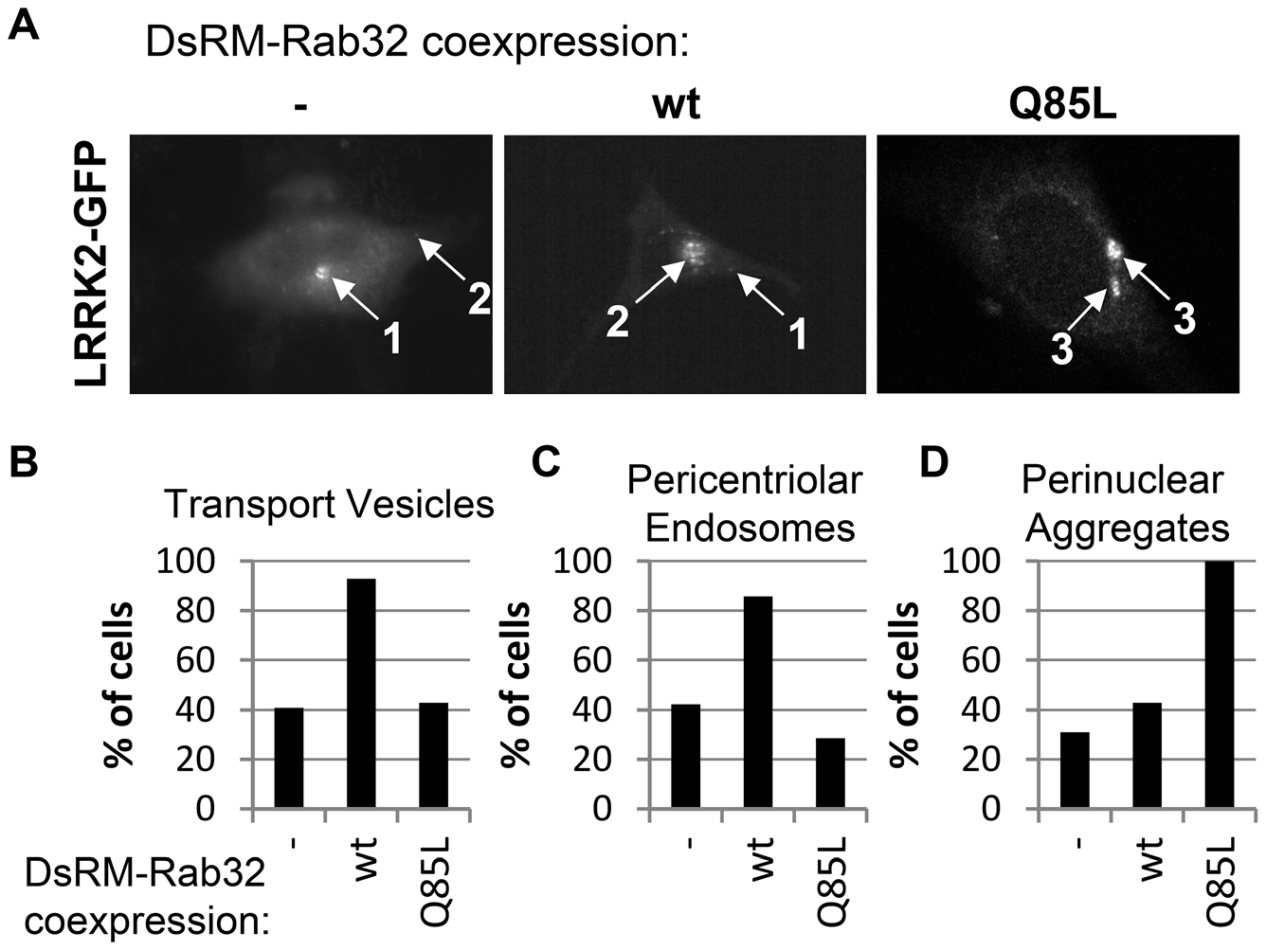
LRRK2-GFP subcellular localization in DsRed-Monomer-Rab32 wt and DsRed-Monomer-Rab32 Q85L overexpressing NIH3T3 cells. (A) Graphical representation of the most common subcellular features of LRRK2-GFP in DsRed-Monomer-Rab32 wt or DsRed-Monomer-Rab32 Q85L expressing cells. 1: pericentriolar endosome; 2: transport vesicles; 3: perinuclear aggregates. (B–D) Microscopic analysis of NIH3T3 cells co-transfected with plasmids encoding DsRed-Monomer-Rab32 wt or DsRed-Monomer-Rab32 Q85L and LRRK2-GFP. In every cell the LRRK2-GFP channel was analyzed for the occurrence of the features transport vesicles, pericentriolar endosomes and perinuclear aggregates. (-) LRRK2-GFP alone: n = 71 cells from 5 independent experiments; LRRK2-GFP and DsRed-Monomer-Rab32 wildtype: n = 15 cells from 3 independent experiments, LRRK2-GFP and DsRed-Monomer-Rab32 Q85L: n = 21 cells from 5 independent experiments.

To quantify the fluorescence microscopic analysis we tested the localization of LRRK2-GFP expressed alone or co-expressed with DsRed-Monomer-Rab32 wt or DsRed-Monomer Rab32 Q85L for the abundance of different features: transport vesicles, pericentriolar recycling endosomes and perinuclear aggregates (Figure 5A). We found that in 50% of total cells expressing LRRK2-GFP only small vesicles occurred. Co-expression of DsRed-Monomer-Rab32 wt with LRRK2-GFP increased this value to 100% of total cells recorded (Figure 5B). In contrast, upon DsRed-Monomer-Rab32 Q85L co-expression with LRRK2-GFP, only a few cells with LRRK2-GFP positive vesicles remained. A similar result was observed for pericentriolar endosomes (Figure 5C). LRRK2-GFP alone displays this typical feature less often (59%) than DsRed-Monomer-Rab32 wt co-expressing cells (92%). Perinuclear aggregates were characteristic for the constitutively active mutant Rab32 Q85L. Looking at LRRK2-GFP this feature occurred in 90% of all DsRed-Monomer-Rab32 Q85L co-expressing cells (Figure 5D). While co-transfected with DsRed-Monomer-Rab32 wt or expressing LRRK2-GFP alone, perinuclear aggregates seemed to be smaller in size and occurred much more infrequently (see also Figure 4C and D). These results show that co-expression of activated or wt Rab32 impacts the cellular localization of LRRK2-GFP in an activity-dependent manner.

**Figure 6:**
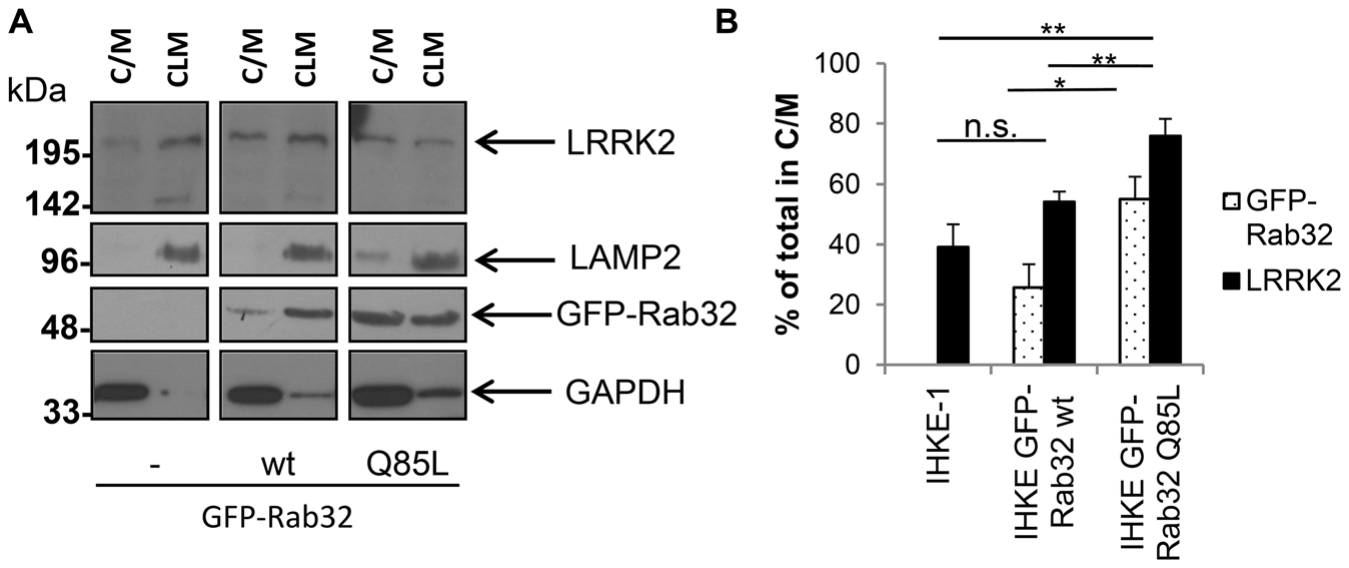
Subcellular fractionation analysis of endogenous LRRK2 in IHKE-1 cells overexpressing GFP-Rab32 wt or GFP-Rab32 Q85L. (A) IHKE-1 cells either untransfected (-) or stably overexpressing GFP-Rab32 wt or GFP-Rab32 Q85L were mechanically disrupted and fractionated by differential centrifugation. Fractions received either contained lysosomes and mitochondria (CLM) or cytosol and light membranes (C/M). Western blot analysis to detect the lysosomal marker LAMP2, GAPDH, Rab32 and LRRK2 were performed with the fractions derived from the different IHKE-1 cells. (B) Quantification of Western blot signal intensities using the gel analyzer functionality of ImageJ. The signal intensity for both the C/M and the CLM fraction was added and set to 100%. The graph shows the % of GFP-Rab32 and endogenous LRRK2 in the C/M fraction of the different cell lines. n = 6 independent experiments. Error bars represent S.E.M.; n.s. = not significant; * = p<0.05; ** = p<0.01.

To verify these data, we performed subcellular fractionation experiments. IHKE-1 cells that either stably expressed GFP-Rab32 wt or GFP-Rab32 Q85L and untransfected IHKE-1 cells as a control were grown on 15 cm diameter cell culture dishes, lysed and crude fractions were prepared. We prepared a fraction which contains lysosomes and mitochondria (CLM = crude lysosome and mitochondria) and a second fraction which contains the cytosol and light membranes like microsomes and endosomes [57]. LAMP2, GAPDH and Rab1B expression detected by Western blotting confirmed that the fractions were relatively pure (Figure 6A and data not shown). We were able to detect GFP-Rab32 wt and GFP-Rab32 Q85L in both fractions, but to a different extent −25.7%±7.7% of GFP-Rab32 wt signal is present in the C/M fraction, while 74.3%±7.7% is in the CLM fraction. By comparison, GFP-Rab32 Q85L is significantly higher in the C/M fraction: 55.1%±7.4% was found in the C/M fraction, but just 44.9%±7.4% in the CLM fraction.

The subcellular distribution of LRRK2 follows the one of Rab32: The C/M fractions displayed an increase in LRRK2 protein content comparing control IHKE-1 cells with GFP-Rab32 wt or GFP-Rab32 Q85L expressing cells and a decrease of LRRK2 protein content in the CLM fraction, respectively (Figure 6A). Quantification of the Western blot signals confirms that in the control cells 39.1%±7.6% of the LRRK2 protein was present in the C/M fraction (60.9%±7.6% in the CLM fraction), for IHKE GFP-Rab32 wt 54.2%±3.4% (45.8%±3.4%) and for IHKE GFP-Rab32 Q85L 75.9%±5.7% (24.1%±5.7%, Figure 6B).

A distinction between late endosomes/MVB and lysosomes upon co-localization analyses of Rab32 Q85L positive perinuclear aggregates and Rab7 in NIH3T3 cells is not possible. This is because Rab7 is found on both types of organelles [55]. The reduction of LRRK2 in the CLM fraction in IHKE GFP-Rab32 Q85L cells together with the observation of increased co-localization of the constitutively active GFP-Rab32 Q85L with Rab7 (but not LAMP2) precludes the possibility that LRRK2 is sorted to lysosomes by Rab32. This is supported by the observation that incubation of neither untransfected IHKE-1 cells nor cells stably transfected with GFP-Rab32 wt with the lysosomal degradation inhibitor Bafilomycin A led to increased LRRK2 levels compared to control cells (Figure S7). Proteins destined for degradation under normal conditions can accumulate upon inhibition of lysosomal degradation via inhibitors. Taken together, these data suggest that the perinuclear aggregates originate from late endosomal structures, and are not associated with lysosomes.

## Discussion

We identified the aminoterminal LRRK2 fragment as a novel Rab32 interacting partner in a yeast two-hybrid screen. Co-immunoprecipitation of GFP-Rab32 or endogenous LRRK2 from IHKE-1 cells confirmed the interaction. Furthermore, pulldown of endogenous LRRK2 from NIH3T3 cells using recombinant GST-Rab32 supported the interaction (Figure 2).

When checking for binding specificity, we found that the LRRK2 interaction is Rab32 and Rab38 specific. Noteworthy, other GTPases tested did not interact with the aminoterminal fragment of LRRK2 that we identified in the yeast two-hybrid screen. Yet another small GTPase, the Rab29 protein (also known as Rab7L1), was recently identified as a new LRRK2 interacting partner [15], [16]. Evolutionarily, Rab29 is most closely related to Rab32 and Rab38. These three GTPases actually belong to a subfamily, based on subtle differences in the generally conserved G-domain, relative to other Rab proteins [42], [58].

In this study we showed that Rab32 binds to the aminoterminal region of LRRK2 whose function is largely unknown so far. Only two domains have previously been described in this region: an ankyrin repeats domain spanning the amino acids 676–902 of human LRRK2, and an armadillo repeats domain from amino acid 49 to 657 [59]. Although the Rab32 and Rab38 interacting partner Varp (or ANKRD27) was shown to bind the GTPases in its ankyrin repeats domain, the Rab32 interacting domain of LRRK2 was located within the armadillo repeats [18]. Because armadillo repeats are known to interact with GTPases, it was speculated, that GTPases are a possible target also for the LRRK2 armadillo repeat domain [60]. In our experiments we could demonstrate that a putative coiled-coil motif within the first 552 aa of human LRRK2 is mandatory for Rab32 binding. Homology modeling of the LRRK2 armadillo repeats domain revealed an extended loop that contains the PD mutation E334K at the position of the putative coiled-coil motif [59], [60]. This loop between armadillo repeat 6 and 7 is part of the Rab32 binding region we identified in this work. Whether this mutation is interfering with the binding capacity to Rab32 remains to be elucidated.

Besides the analysis of Rab32 binding to LRRK2, we could demonstrate the co-localization of LRRK2 and wildtype Rab32 at transport vesicles and recycling endosomes and for LRRK2 and the constitutively active Rab32 mutant at late endosomes/MVB. In live cell imaging experiments we observed several sorting events, thus demonstrating co-transport of Rab32 and LRRK2. It has previously been shown that LRRK2 co-localizes with endosome markers such as Rab7 and Rab5B, or the endosomal transport marker γ-adaptin [6], [8], [11], [12], [13], [14]. The latter was part of the AP-1 adapter protein complex, which was recently demonstrated to directly interact with Rab32 [24]. This interaction plays a role in Rab32 mediated endosomal transport of LRO containing cells like melanocytes or platelets [17], [24], [25]. Even more, the AP-1 complex subunit β-1 was found in a GST-pulldown based screen to directly interact with LRRK2 [61]. Together with our own observations these findings suggest a role for Rab32 in LRRK2 late endosomal transport and sorting.

Constitutively activated Rab32 showed increased co-localization with the late endosome/MVB marker Rab7 in IHKE-1 cells or Rab7 positive perinuclear aggregates in NIH3T3 cells. We demonstrated that overexpression of constitutively active Rab32 led to an increased size of perinuclear aggregates, an observation also made by another group upon LRRK2 G2019S overexpression [11]. Here, the authors came to the conclusion, that LRRK2 influences the Rab7 dependent lysosome positioning in the cell. Several groups have demonstrated that small GTPases seemed to be responsible for LRRK2 localization changes in cells [8], [9], [15]. Our data also support a role for Rab32 in LRRK2 intracellular localization. We could not only demonstrate that Rab32 Q85L overexpression led to the translocation of LRRK2-GFP to the Rab7 positive perinuclear aggregates, but also that Rab32 Q85L induced the formation of these structures. The precise mechanism remains to be elucidated in future work.

Activated Rab32 did not simply seem to guide LRRK2 to lysosomes. We treated GFP-Rab32 stably expressing IHKE-1 cells with the lysosomal degradation inhibitor bafilomycin A. If Rab32 increases LRRK2 lysosomal degradation in GFP-Rab32 overexpressing cells, we would expect accumulation of LRRK2 upon Bafilomycin A treatment, but this was not the case. Furthermore, subcellular fractionation revealed that GFP-Rab32 overexpression and expression of constitutively active Rab32 removed LRRK2 from the lysosomes and mitochondria containing fraction. This indicates that the Rab7 positive aggregates of co-localized LRRK2 with Rab32 Q85L represent enlarged late endosomes or MVBs.

In addition to the function of Rab32 in cells containing LROs, a role for Rab32 in regulating mitochondria has been identified [17], [29]. Rab32 was found at the mitochondria-ER interface, where it was demonstrated to act as PKA anchoring protein [27], [62]. PKA in turn was shown to phosphorylate LRRK2 which is then mediating cytotoxic effects that were assumed to play a role in the pathogenesis of PD [63], [64]. LRRK2 is involved in mitochondrial dysfunction, which is discussed as a cause of Parkinson's disease [65]. Our fractionation experiments showed that Rab32 recruited LRRK2 away from the lysosome and mitochondria containing fraction in IHKE-1 cells upon overexpressing wildtype or constitutively active Rab32. Although we focused on the role of Rab32 in LRRK2 LE/lysosomal localization it cannot be excluded, that activated or overexpressed GFP-Rab32 targets LRRK2 away from mitochondria and mediates its transport to late endosomes/MVB.

Taken together our results describe Rab32 as a novel LRRK2 interacting protein controlling its late endosomal trafficking, implying a novel role in regulation of the LRRK2 protein. A distinct role of the Rab32 GTPase in PD remains still illusive. It was shown, that Rab32 is present in brain, but the expression is very low under normal conditions [43], [58]. It has recently been demonstrated, that Rab32 mRNA levels increase in mouse brains after stimulation with LPS [32]. Whether there is an elevated Rab32 expression under pathophysiological conditions, like PD, remains to be elucidated. For the small GTPases Rab5B, Rab7 and RAB7L1 (Rab29), a regulatory role on LRRK2 in PD has recently been described [8], [15], [16]. Furthermore, there is evidence, that LRRK2 itself plays a role in late endocytic transport pathways. It could be demonstrated, that the PD mutation LRRK2 G2019S decreased the transport of EGF in a Rab7 mediated manner [66]. Similar to the Rab32 regulation of late endosomal transport processes, the LRRK2 interacting GTPase Rab7L1 regulates late endosomal transport of LRRK2, as well [15]. Therefore, a role for Rab32 in processes underlying the pathophysiology of PD is definitely possible making it to a promising target for further functional studies.

## Supporting Information

Figure S1 **Expression of endogenous Rab32 and GFP-Rab32 wt in control IHKE-1 and stably transfected IHKE-1 (IHKE GFP-Rab32 wt) cells.** Equal amounts of cell lysate from IHKE-1 and IHKE GFP-Rab32 wt were subjected to SDS-PAGE followed by Western blotting. Rab32 was detected with an anti-Rab32 antibody.
(TIF)

Figure S2 **Coiled-coil motif prediction within LRRK2 residues 1–552.** The first 552 amino acids of LRRK2 were entered to the coils algorithm that calculates the probability of coiled-coil motifs. The image detects a high probability of such a structure between the amino acids 319–348.
(TIF)

Figure S3 **Co-localization analysis of DsRed-Monomer-Rab32 wt and DsRed-Monomer-Rab32 Q85L with GFP-Rab5A and GFP-Rab32 wt and GFP-Rab32 Q85L with endogenous Rab9.** (A) For Rab5 co-localization analysis NIH3T3 cells were co-transfected with plasmids encoding for DsRed-Monomer-Rab32 wt or DsRed-Monomer-Rab32 Q85L and the early endosomal marker protein GFP-Rab5A. Cells were fixed in 4% PFA and subjected to microscopic analyzes. Scale bar = 10 µm. (B) For Rab9 co-localization analysis NIH3T3 cells were transfected with the indicated GFP-Rab32 wt or GFP-Rab32 Q85L expression plasmids. 24 hours after transfection cells were fixed and subjected to secondary immunofluorescence staining of Rab9. Scale bar = 10 µm.
(TIF)

Figure S4 **Co-localization analysis of LRRK2-GFP with Rab7 and Rab11.** For co-localization analysis NIH3T3 cells were transfected with plasmids encoding for LRRK2-GFP and immunostained for Rab7 or Rab11 localization, respectively. Cells were fixed in 4% PFA and subjected to microscopic analyzes. Scale bar = 10 µm.
(TIF)

Figure S5 **Golgi and endosomal co-localization of GFP-Rab32 wt.** IHKE-1 cells were transiently transfected with pEGFP-Rab32 wt. After 24 hours cells were fixed and immuno-stained for the indicated proteins. Scale bar = 10 µm.
(TIF)

Figure S6 **Analysis of GFP-Rab32 wt and GFP-Rab32 Q85L co-localization with Rab7 and LAMP2 in IHKE-1 cells.** (A) IHKE GFP-Rab32 wt and IHKE GFP-Rab32 Q85L cells were grown on glass cover slips, fixed and stained for Rab7 by secondary immunofluorescence. Scale bar = 10 µm. (B) IHKE GFP-Rab32 wt and IHKE GFP-Rab32 Q85L cells were grown on glass cover slips, fixed and stained for LAMP2 by secondary immunofluorescence. Scale bar = 10 µm.
(TIF)

Figure S7 **LRRK2 expression in untransfected (IHKE-1) and IHKE GFP-Rab32 wt cells upon Bafilomycin A treatment.** Western blots of endogenous LRRK2 and LC3B of IHKE-1 and stably GFP-Rab32 wt expressing IHKE-1 cells. Cells were grown for 24 hours. After incubation with 100 nM Bafilomycin A for another 24 hours, cells were lysed and the proteins separated by SDS-PAGE followed by subsequent Western blot analysis. n = 3 independent experiments.
(TIF)

Movie S1 **Live cell imaging of DsRed-Monomer-Rab32 wt and LRRK2-GFP.** NIH3T3 cells were grown in live cell microscopy chambers for 24 hours. Then, cells were transfected with plasmids encoding DsRed-Monomer-Rab32 wt and LRRK2-GFP. After 48 hours of incubation living cells were imaged using a Zeiss LSM5 live microscope. Images were captured in live mode every second, both channels simultaneously.
(MP4)

Movie S2 **Live cell imaging of DsRed-Monomer-Rab32 wt and LRRK2-GFP.** NIH3T3 cells were grown in live cell microscopy chambers for 24 hours. Then, cells were transfected with plasmids encoding DsRed-Monomer-Rab32 wt and LRRK2-GFP. After 48 hours of incubation living cells were imaged using a Zeiss LSM5 live microscope. Images were captured in live mode every second, both channels simultaneously.
(MP4)
