# Supplementary figures and images for: LRRK2 Transport Is Regulated by Its Novel Interacting Partner Rab32
Source: PLoS One. 2014 Oct 31;9(10):e111632. doi: 10.1371/journal.pone.0111632 (PMC4216093; doi:10.1371/journal.pone.0111632)

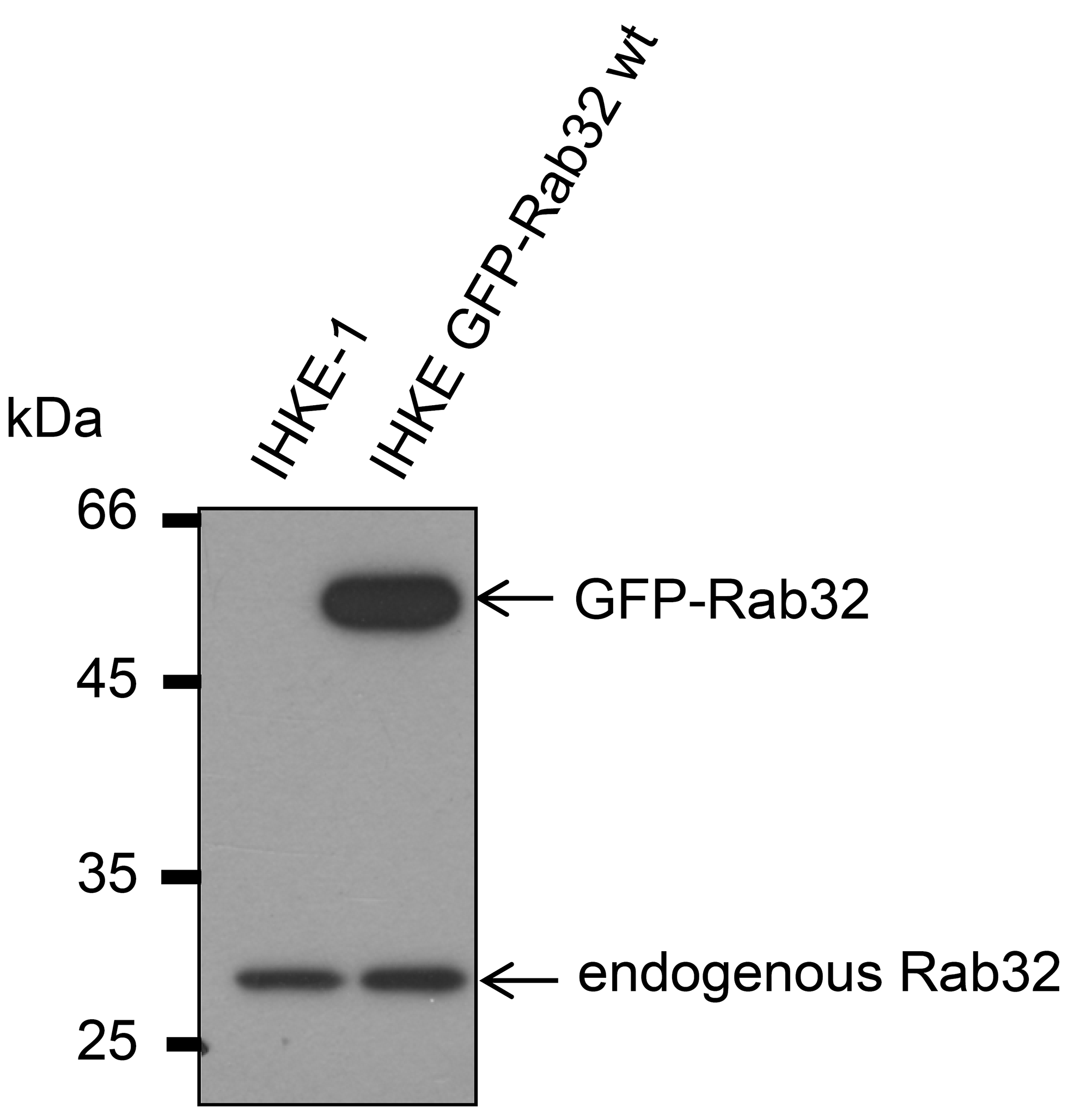

Supplement: Figure S1 — Expression of endogenous Rab32 and GFP-Rab32 wt in control IHKE-1 and stably transfected IHKE-1 (IHKE GFP-Rab32 wt) cells. Equal amounts of cell lysate from IHKE-1 and IHKE GFP-Rab32 wt were subjected to SDS-PAGE followed by Western blotting. Rab32 was detected with an anti-Rab32 antibody. (TIF) [file pone.0111632.s001.tif]

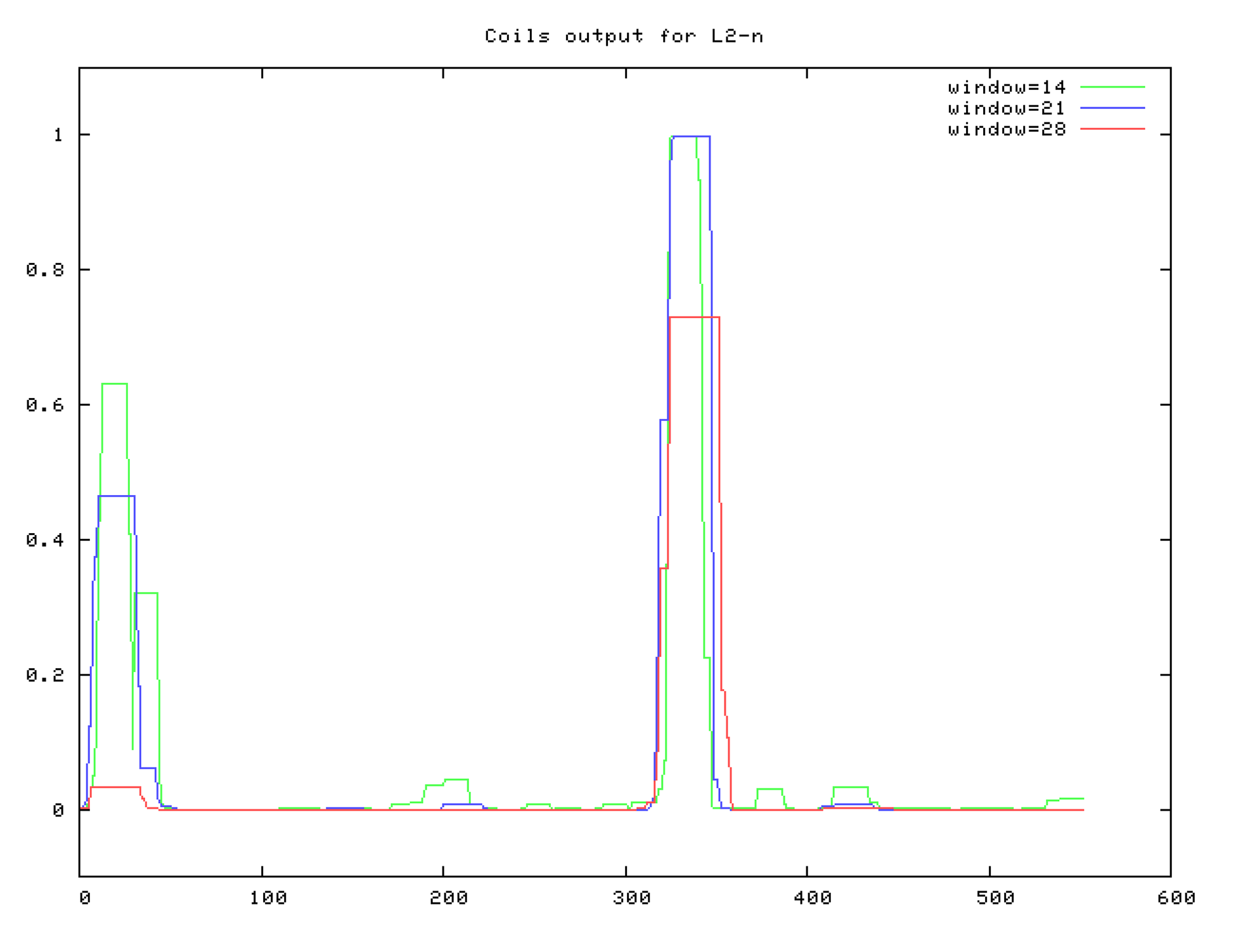

Supplement: Figure S2 — Coiled-coil motif prediction within LRRK2 residues 1–552. The first 552 amino acids of LRRK2 were entered to the coils algorithm that calculates the probability of coiled-coil motifs. The image detects a high probability of such a structure between the amino acids 319–348. (TIF) [file pone.0111632.s002.tif]

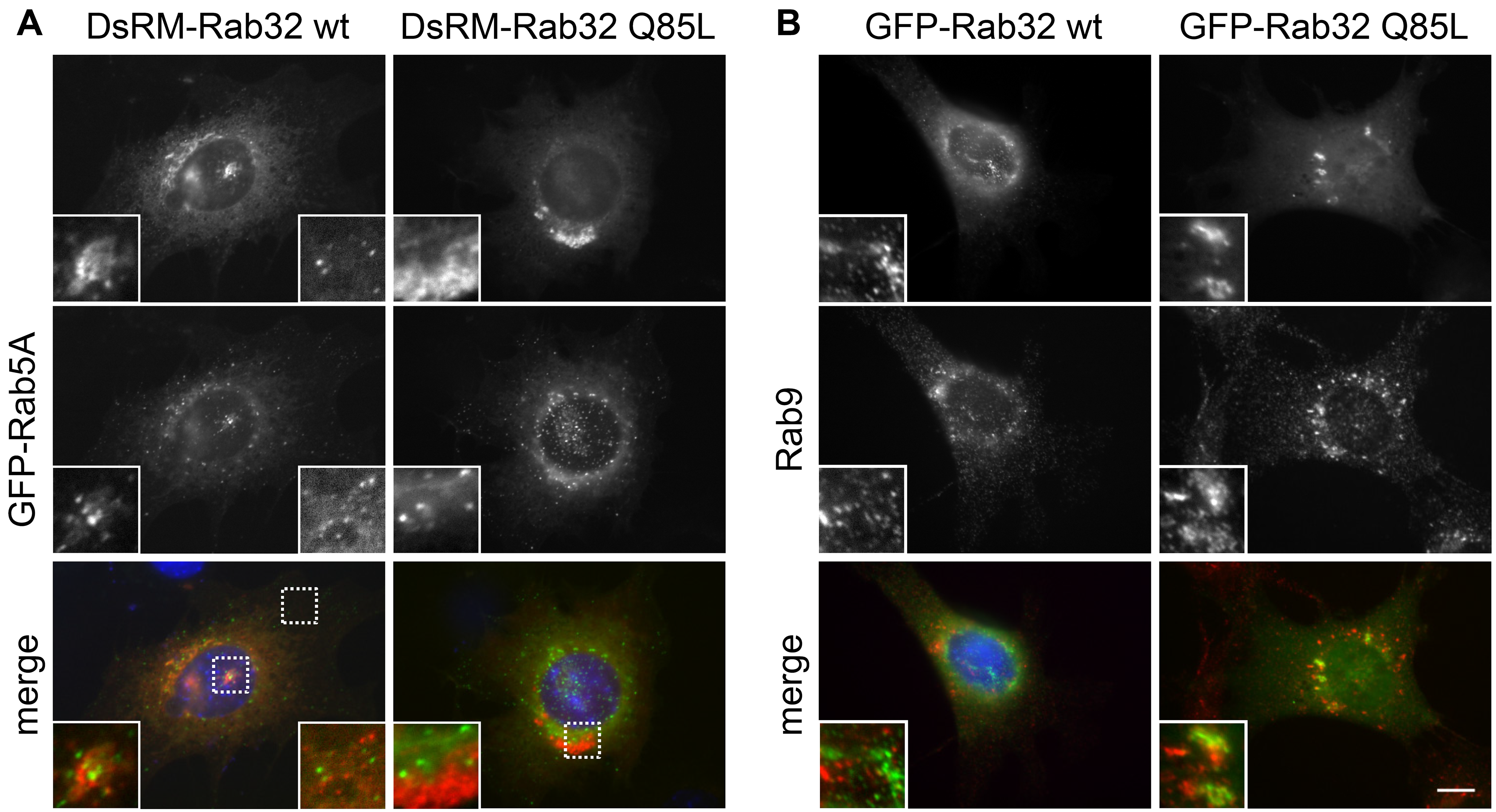

Supplement: Figure S3 — Co-localization analysis of DsRed-Monomer-Rab32 wt and DsRed-Monomer-Rab32 Q85L with GFP-Rab5A and GFP-Rab32 wt and GFP-Rab32 Q85L with endogenous Rab9. (A) For Rab5 co-localization analysis NIH3T3 cells were co-transfected with plasmids encoding for DsRed-Monomer-Rab32 wt or DsRed-Monomer-Rab32 Q85L and the early endosomal marker protein GFP-Rab5A. Cells were fixed in 4% PFA and subjected to microscopic analyzes. Scale bar = 10 µm. (B) For Rab9 co-localization analysis NIH3T3 cells were transfected with the indicated GFP-Rab32 wt or GFP-Rab32 Q85L expression plasmids. 24 hours after transfection cells were fixed and subjected to secondary immunofluorescence staining of Rab9. Scale bar = 10 µm. (TIF) [file pone.0111632.s003.tif]

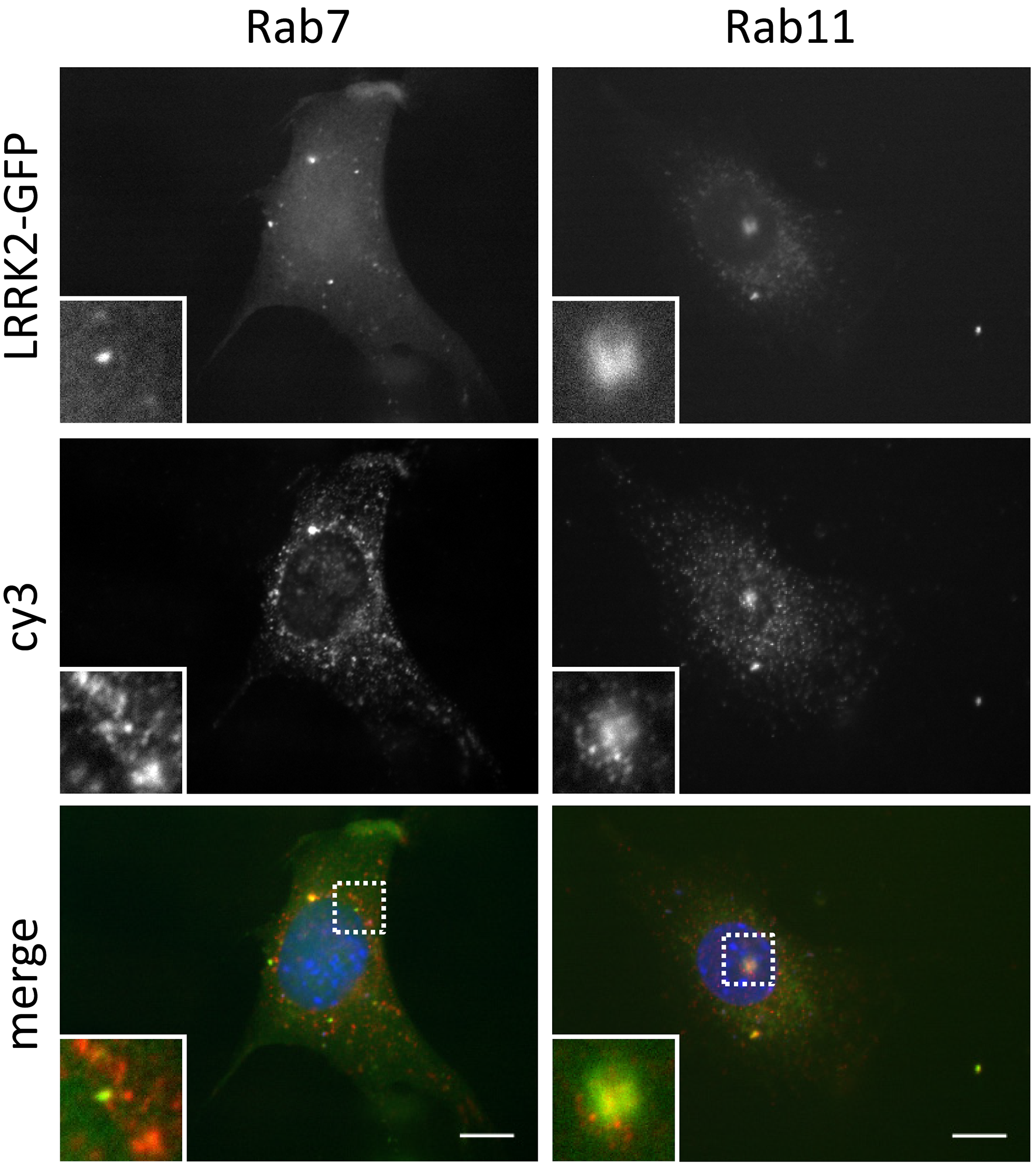

Supplement: Figure S4 — Co-localization analysis of LRRK2-GFP with Rab7 and Rab11. For co-localization analysis NIH3T3 cells were transfected with plasmids encoding for LRRK2-GFP and immunostained for Rab7 or Rab11 localization, respectively. Cells were fixed in 4% PFA and subjected to microscopic analyzes. Scale bar = 10 µm. (TIF) [file pone.0111632.s004.tif]

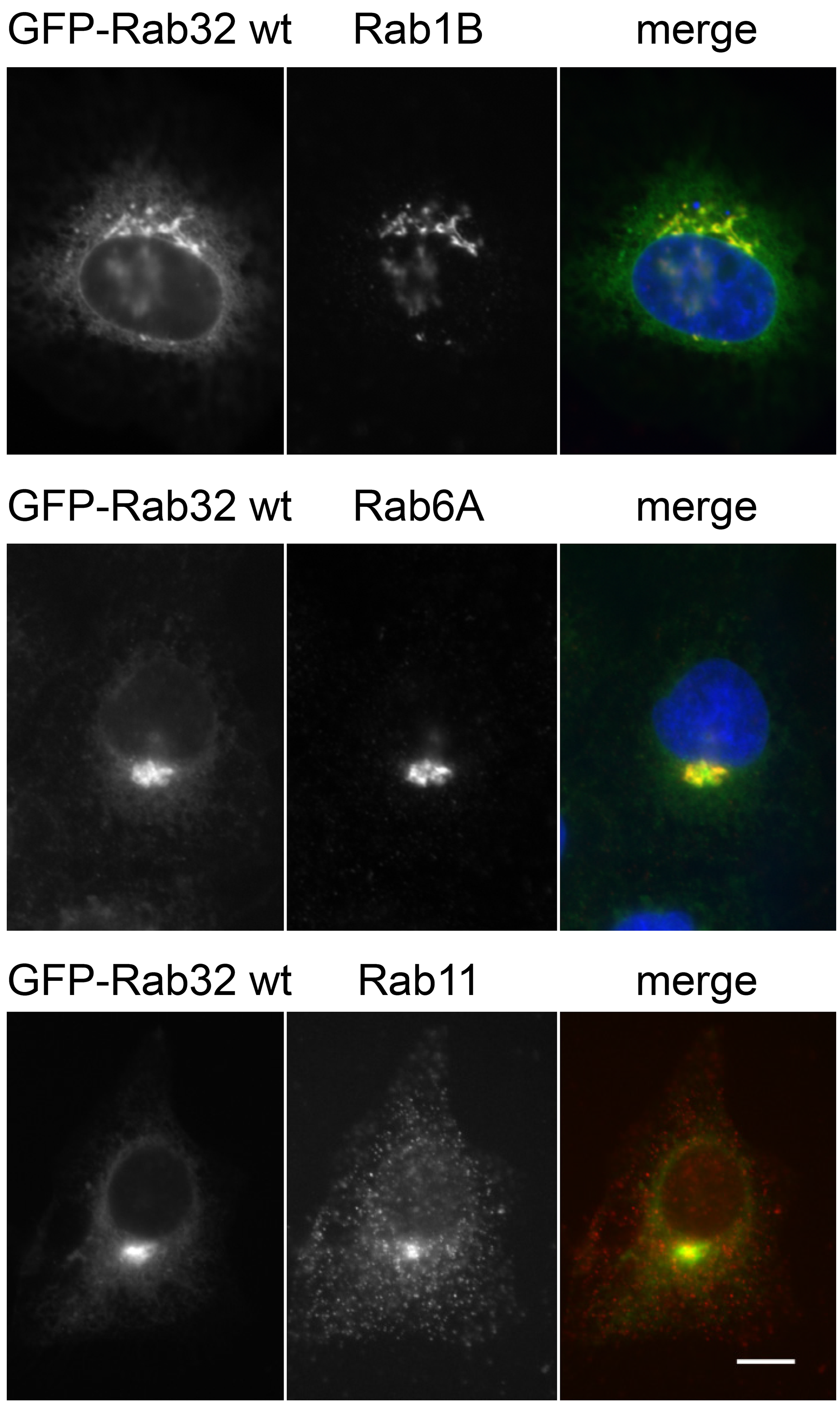

Supplement: Figure S5 — Golgi and endosomal co-localization of GFP-Rab32 wt. IHKE-1 cells were transiently transfected with pEGFP-Rab32 wt. After 24 hours cells were fixed and immuno-stained for the indicated proteins. Scale bar = 10 µm. (TIF) [file pone.0111632.s005.tif]

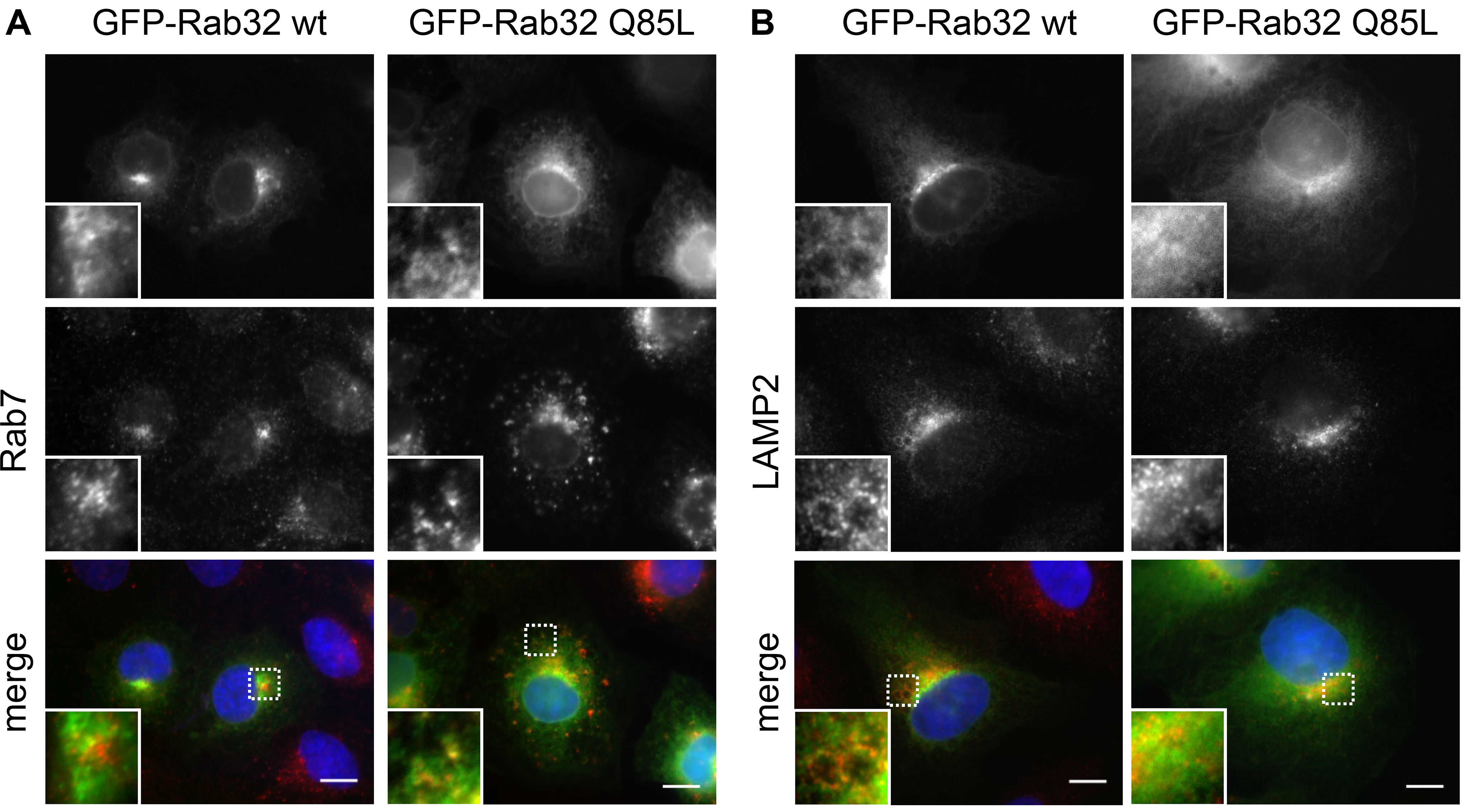

Supplement: Figure S6 — Analysis of GFP-Rab32 wt and GFP-Rab32 Q85L co-localization with Rab7 and LAMP2 in IHKE-1 cells. (A) IHKE GFP-Rab32 wt and IHKE GFP-Rab32 Q85L cells were grown on glass cover slips, fixed and stained for Rab7 by secondary immunofluorescence. Scale bar = 10 µm. (B) IHKE GFP-Rab32 wt and IHKE GFP-Rab32 Q85L cells were grown on glass cover slips, fixed and stained for LAMP2 by secondary immunofluorescence. Scale bar = 10 µm. (TIF) [file pone.0111632.s006.tif]

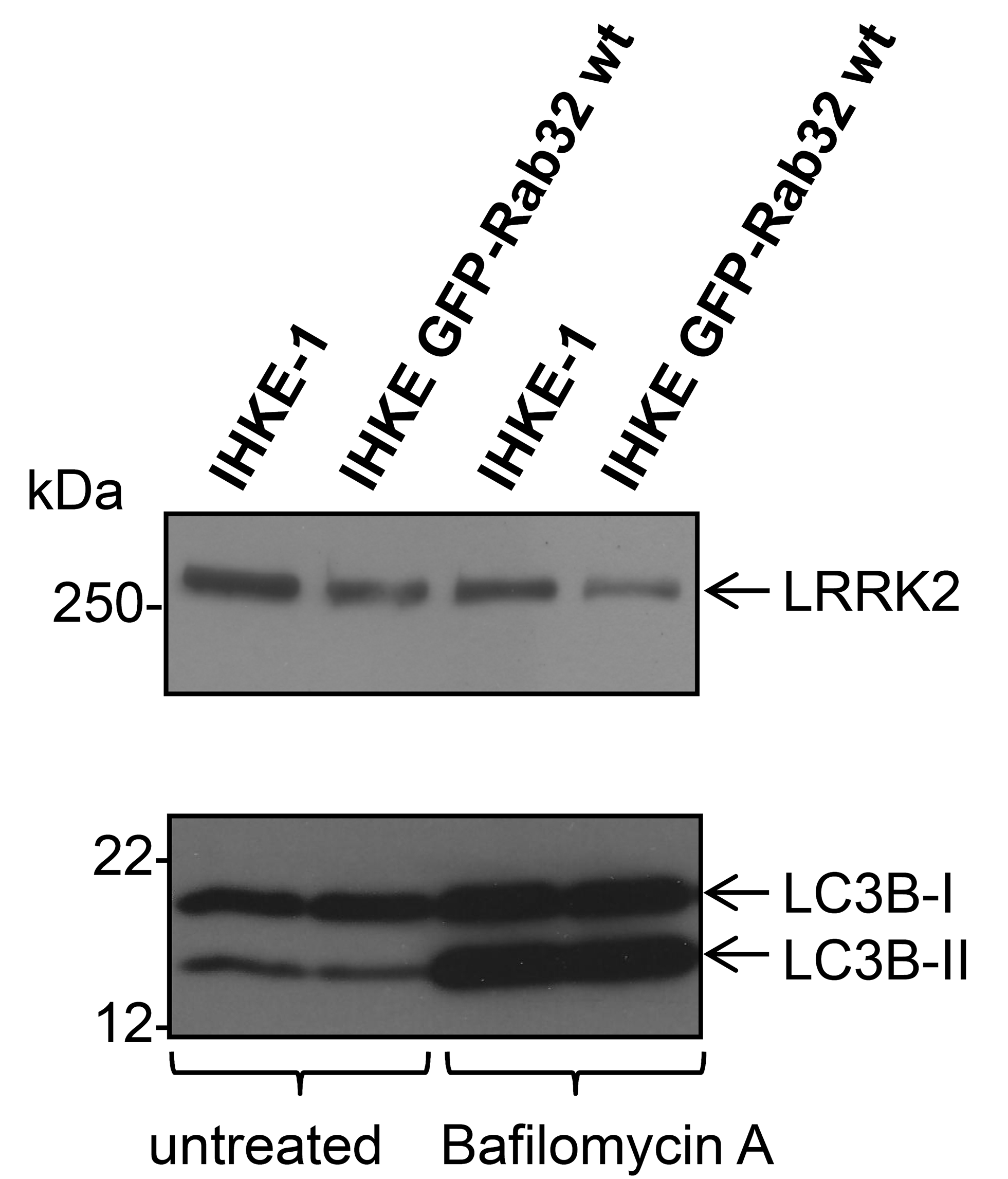

Supplement: Figure S7 — LRRK2 expression in untransfected (IHKE-1) and IHKE GFP-Rab32 wt cells upon Bafilomycin A treatment. Western blots of endogenous LRRK2 and LC3B of IHKE-1 and stably GFP-Rab32 wt expressing IHKE-1 cells. Cells were grown for 24 hours. After incubation with 100 nM Bafilomycin A for another 24 hours, cells were lysed and the proteins separated by SDS-PAGE followed by subsequent Western blot analysis. n = 3 independent experiments. (TIF) [file pone.0111632.s007.tif]
